# Primary Renal Synovial Sarcoma: A Rare Case Presentation

**DOI:** 10.7759/cureus.72256

**Published:** 2024-10-24

**Authors:** Mansoor Abbas, Muhammad Awais Majeed, Maryyam Liaqat, Shehar Bano, Tuba Khalid, Nahel Chaudhry, Faisal Qayum, Shahzeb Ahmed, Amna Karim, Farzouq Javaid

**Affiliations:** 1 Medical Oncology, Shaukat Khanum Memorial Cancer Hospital and Research Centre, Lahore, PAK; 2 Internal Medicine, King Edward Medical University, Lahore, PAK

**Keywords:** chemotherapy, leiomyosarcoma, sarcoma, sarcomatoid, synovial

## Abstract

Synovial sarcoma is a rare mesenchymal tumor, and its occurrence as a primary renal tumor is exceedingly rare. We are presenting a case of renal synovial sarcoma with lung involvement in a 47-year-old female patient who initially presented with typical renal symptoms, including blood in urine and left flank pain. Imaging revealed a large renal mass with extension into the renal vein and metastatic nodules in the lungs. Histopathological examination and genetic analysis confirmed monophasic synovial sarcoma with SYT-SSX2 translocation. Treatment included radical nephrectomy followed by systemic chemotherapy with doxorubicin and ifosfamide. Despite the initial response, the disease progressed, leading to fatal complications. This case highlights the diagnostic challenges, limited treatment options, and poor prognosis associated with primary renal synovial sarcoma.

## Introduction

Synovial sarcoma is a rare tumor of mesenchymal origin and accounts for only 6-10% of soft tissue tumors; it more commonly originates near the extremities of the limbs in children and young adults [1]. Primary renal sarcoma is a rare soft tissue sarcoma comprising only 1% of all renal tumors [2]. In early 2000, this type of rare sarcoma, initially considered embryonal sarcoma, was first described by Argani et al. [3]. It is a rare tumor that harbors a poor prognosis and can only be diagnosed with immunohistochemistry and genetic analysis. Genomic sequencing should be considered in cases of uncertain diagnosis [3,4]. The literature review revealed a significant number of cases of renal synovial sarcoma; these cases have received variable treatments and have varying outcomes. We present here a case of a patient who had hematuria and left flank pain. She underwent a nephrectomy for her left renal tumor. The histopathology report of the surgical specimen and genomic sequencing revealed it to be a synovial sarcoma of renal origin, and imaging provided evidence of lung metastasis.

## Case presentation

**Figure 1:**
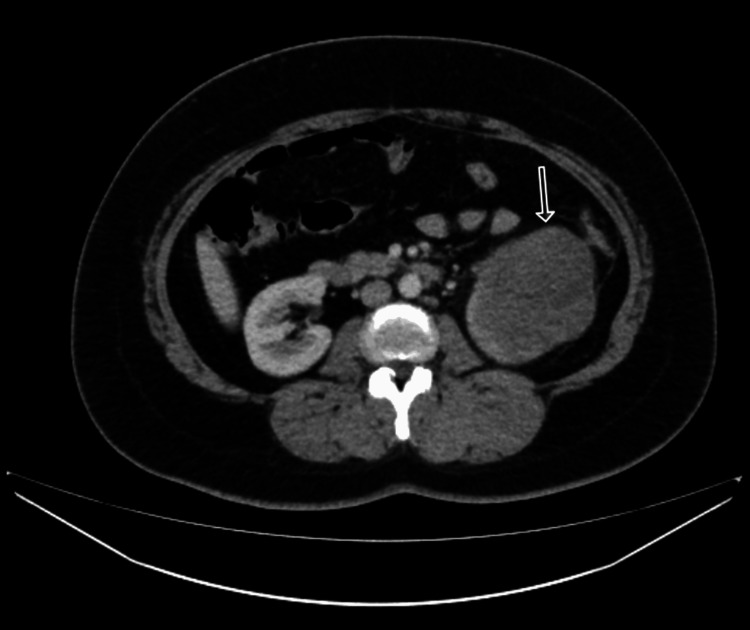
CT abdomen and pelvis with contrast showing an exophytic heterogeneously enhancing mass in the left renal lower pole.

A 47-year-old female patient presented with a two-month history of gross blood in her urine and left-sided abdominal pain in the lumbar region. Abdominal examination revealed a mass in the left upper abdomen. An ultrasound abdomen and pelvis from an outside hospital showed a mass in the left lumber region. A computerized tomography (CT) scan revealed an exophytic mass measuring approximately 8.9 cm × 7.9 cm × 10.9 cm, which was heterogeneously enhanced in the left renal lower pole and extended into the left renal vein (Figure 1).

**Figure 2:**
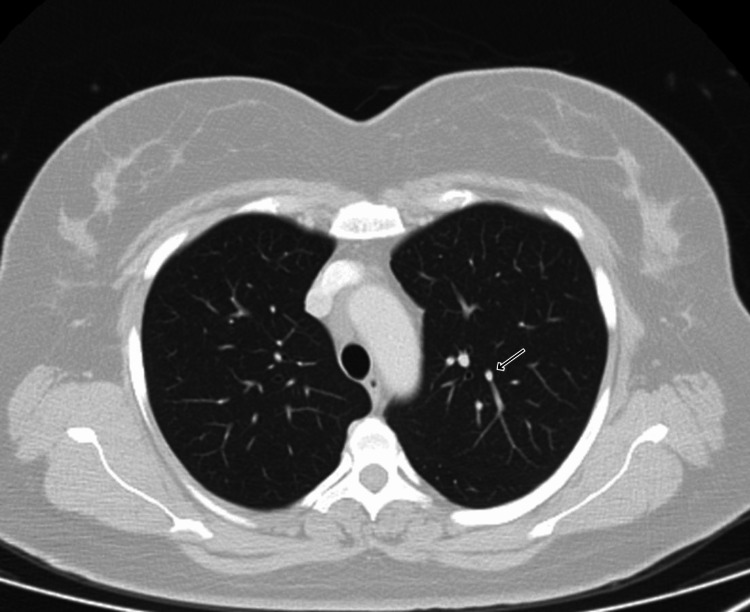
CT chest showing multiple pulmonary nodules.

We also observed multiple lung nodules in the anterior segment of the left lobe, measuring 8 mm and 6 mm in the right middle lobe, and 10-mm nodules in the lateral segment of the right middle lobe, indicating lung metastases and classifying the disease as stage IV (Figure 2).

**Figure 3:**
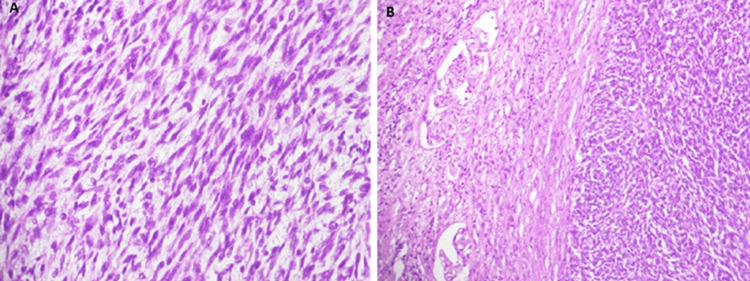
(A) This section shows a malignant tumor made up of spindled tumor cells arranged in herring-bone and fascicular patterns. The tumor has high cellularity and moderate cytological atypia. 10-12 mitosis are seen per 10 HPFs. (B) This section shows the interface of the tumor with the renal parenchyma.

The case was discussed in a urology multidisciplinary team meeting, and a provisional diagnosis of renal cell carcinoma with lung metastasis was made. The patient underwent a left radical nephrectomy. Intraoperative findings suggested a left renal exophytic tumor with a thrombus extending to the inferior vena cava. Histopathological examination revealed a malignant spindle cell neoplasm of the left kidney involving the renal sinus fat. Capsule, perinephric fat, ureteric, and vascular margins were free of tumors (Figure 3A-3B).

**Figure 4:**
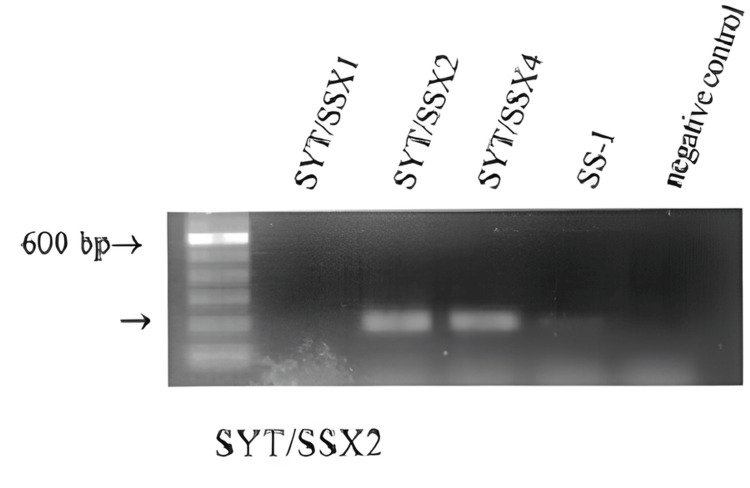
Standard SYT/SSX2 PCR -band size 10 kb. SYT/SSX2 RT-PCR reaction was carried out using specific oligonucleotide primers for SYT/SSX2. Using an optimized nested RT-PCR amplification that includes outer and inner oligonucleotide primers specific for SYT sequences located far upstream from the fusion gene breakpoint, an outer SSX oligonucleotide primer specific for a sequence in the untranslated region, and an inner oligonucleotide primer at the 3′ end of the SSX involved in the fusion gene led to a robust detection of specific SYT/SSX fusion transcript.

Immunohistochemistry was positive for PAX 8 and SS18. Monophasic synovial sarcoma and sarcomatoid carcinoma were considered in the differential diagnosis. Fluorescent in situ hybridization (FISH) for SS18 was suggested to evaluate synovial sarcoma. FISH analysis confirmed presence of SYT-SSX2 (X, 18) translocation (Figure 4).

**Figure 5:**
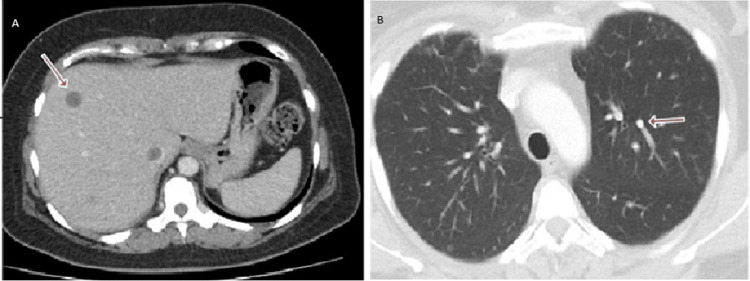
CT scan showing (A) Interval reduction in size of hepatic metastasis; (B) stable pulmonary metastasis.

Given the stage 4 nature of the disease, we initiated systemic chemotherapy. The patient received two cycles of doxorubicin, but the disease progressed clinically and radiologically, so they switched to ifosfamide. A CT scan after four cycles of ifosfamide showed an interval decrease in the size of hepatic metastasis and stable pulmonary metastasis (Figure 5A-5B).

**Figure 6:**
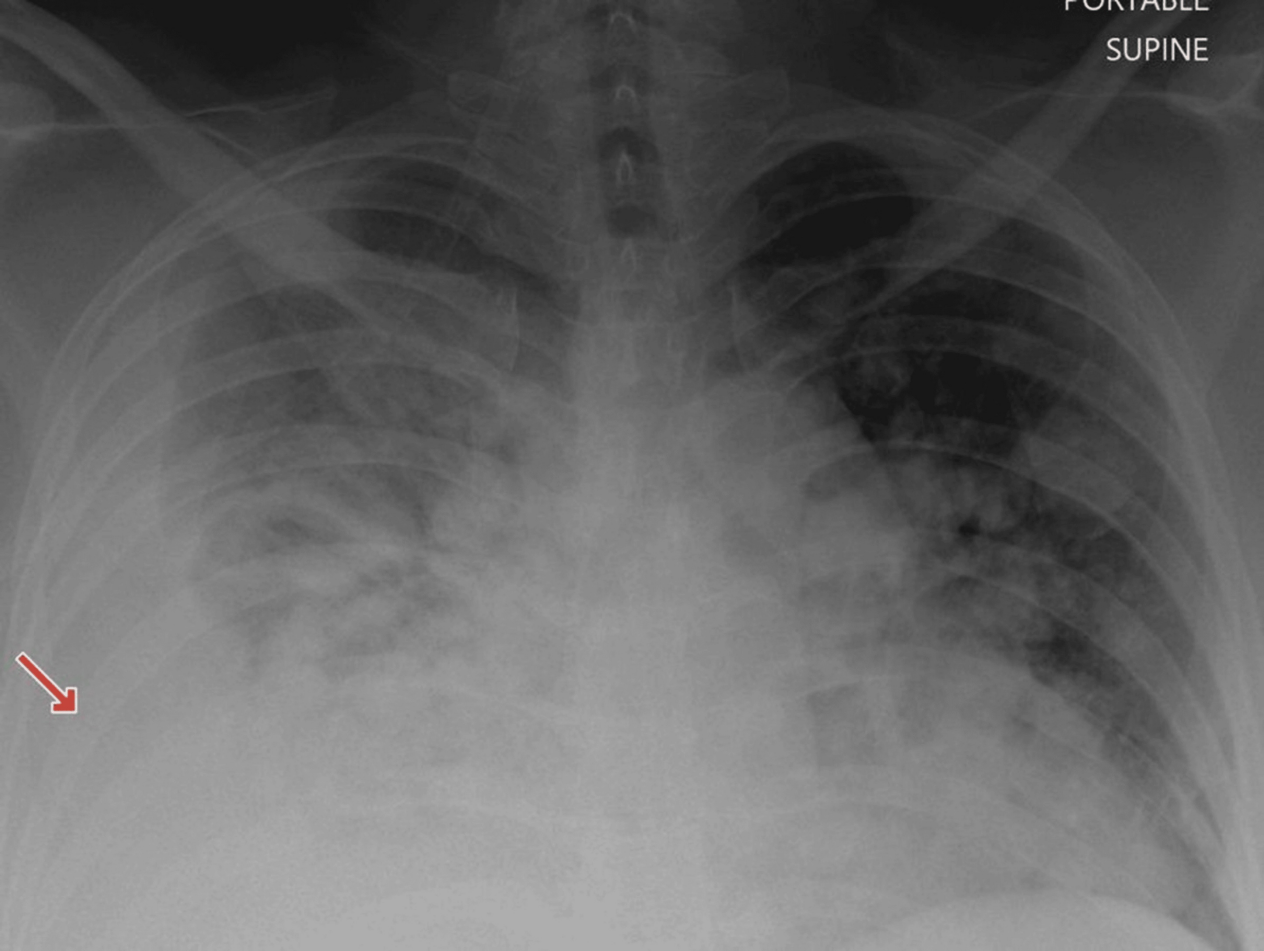
X-ray chest PA view showing interval development of moderate right sided pleural effusion.

The patient received eight cycles of ifosfamide till August 2023. A post-chemotherapy scan in October 2023 showed stable disease. Later, she presented in an emergency with complaints of shortness of breath, and a chest X-ray showed moderate pleural effusion (Figure 6).

A CT CAP was planned to assess the disease status, but unfortunately, the patient did not survive.

## Discussion

The most common histology of renal sarcoma is leiomyosarcoma; almost 40-60% of renal sarcoma is morphologically leiomyosarcoma [4]. Some other types of renal sarcoma have been described in the literature, which are less common than leiomyosarcoma [2]. A rare type of sarcoma is synovial, which is of mesenchymal origin and exhibits specific chromosomal translocation of t(X,18) (p11; q11). It can easily be confused with renal cell carcinoma due to its similar presentation [5,6]. Common sites of synovial sarcoma are body extremities, but less commonly they can also occur in serous surfaces, soft tissue and walls of body cavities, and head and neck structures [7].

Primary renal sarcoma is encountered quite rarely, and while making the diagnosis and reviewing the histopathology of the tissue specimen from suspected cases, differentials of sarcomatous renal and retroperitoneal sarcomas with secondary renal involvement should be considered [2].

Histopathology and immunocytochemistry help diagnose most of these tumors. Genetic analysis should be considered in cases where the diagnosis remains uncertain.

Primary renal soft tissue sarcomas primarily affect adults aged 15 to 71 years, exhibiting an equal incidence in both genders and presenting with abdominal pain and hematuria. Owing to their aggressive nature, these tumors carry a poor prognosis [3,4]. Translocation (X; 18) (p11.2; q11.2) will lead to synovial sarcoma development. Fusion of the SYT gene on chromosome 18 with one of three homologous genes, SSX1, SSX2, and SSX4, on the X chromosome results in pathological tissue development of synovial sarcoma [8].

Other than these cytogenetic changes, another general constitutive alteration of synovial sarcomas is Bcl-2 expression, as assessed by immunohistochemistry [9]. The general treatment protocol of primary renal synovial sarcoma comprises radical nephrectomy with adjuvant Ifosfamide and Doxorubicin-based chemotherapy regimens [10]. Blas and Roberti reported 113 patients with a median follow-up of 12 months; the median disease-free survival was 25 months; and overall survival was 34 months. The recurrence rate was 39.8%. The risk of death was increased with metastasis at diagnosis and the patients who had recurrence. The female population had better survival outcomes [11].

We have not reached a consensus on appropriate chemotherapy regimens for treatment after reviewing the literature, necessitating further clinical trials to establish a definitive treatment protocol for the rare primary renal synovial sarcoma.

## Conclusions

Primary renal synovial sarcoma is one of the rare entities we encounter while dealing with renal sarcomas, thorough histopathological and genetic analysis should be done to rule out other rare entities and establish the proper diagnosis. Currently, surgery followed by chemotherapy is being used as a treatment option for this tumor. Still, we need to establish definite treatment protocols by incorporating these patients in clinical trials and establishing better treatment options. Other options for targeted therapies and the role of immunotherapy should also be explored. This will help us to improve the outcome of patients with this rare tumor.
